# Texture Analysis of Dynamic Contrast-Enhanced MRI in Evaluating Pathologic Complete Response (pCR) of Mass-Like Breast Cancer after Neoadjuvant Therapy

**DOI:** 10.1155/2019/4731532

**Published:** 2019-12-26

**Authors:** Kun Cao, Bo Zhao, Xiao-Ting Li, Yan-Ling Li, Ying-Shi Sun

**Affiliations:** Department of Radiology, Key Laboratory of Carcinogenesis and Translational Research, Ministry of Education, Peking University Cancer Hospital and Institute, Beijing, China

## Abstract

**Objectives:**

MRI is the standard imaging method in evaluating treatment response of breast cancer after neoadjuvant therapy (NAT), while identification of pathologic complete response (pCR) remains challenging. Texture analysis (TA) on post-NAT dynamic contrast-enhanced (DCE) MRI was explored to assess the existence of pCR in mass-like cancer.

**Materials and Methods:**

A primary cohort of 112 consecutive patients (40 pCR and 72 non-pCR) with mass-like breast cancers who received preoperative NAT were retrospectively enrolled. On post-NAT MRI, volumes of the residual-enhanced areas and TA first-order features (19 for each sequence) of the corresponding areas were achieved for both early- and late-phase DCE using an in-house radiomics software. Groups were divided according to the operational pathology. Receiver operating characteristic curves and binary logistic regression analysis were used to select features and achieve a predicting formula. Overall diagnostic abilities were compared between TA and radiologists' subjective judgments. Validation was performed on a time-independent cohort of 39 consecutive patients.

**Results:**

TA features with high consistency (Cronbach's alpha >0.9) between 2 observers showed significant differences between pCR and non-pCR groups. Logistic regression using features selected by ROC curves generated a synthesized formula containing 3 variables (volume of residual enhancement, entropy, and robust mean absolute deviation from early-phase) to yield AUC = 0.81, higher than that of using radiologists' subjective judgment (AUC = 0.72), and entropy was an independent risk factor (*P* < 0.001). Accuracy and sensitivity for identifying pCR were 83.93% and 70.00%. AUC of the validation cohort was 0.80.

**Conclusions:**

TA may help to improve the diagnostic ability of post-NAT MRI in identifying pCR in mass-like breast cancer. Entropy, as a first-order feature to depict residual tumor heterogeneity, is an important factor.

## 1. Introduction

Neoadjuvant therapy (NAT) is an essential procedure in breast cancer treatment. Whether a pathologic complete response (pCR) can be achieved represents a prognostic factor that is related to tumor recurrence and survival [1]. Proper evaluation of residual tumor tissue after NAT can help clinicians optimize NAT while avoiding unnecessary therapy. Magnetic resonance imaging (MRI) is considered the best approach with the highest accuracy to evaluate such residual tumors [2–4].

However, imaging appearances of residual tumors after treatment are different from those at pretreatment, thus making them difficult to evaluate. The only criterion currently used on MRI is enhancement [5, 6]. Only when no enhancement is observed at the corresponding primary tumor site can a conclusion of radiological complete response (iCR) be drawn. Nevertheless, both false positives and false negatives occur. According to a meta-analysis based on 25 studies [7], the specificity of MRI in predicting pCR was as high as 90.7%, but the sensitivity was only 63.1%. Hence, such criterion lacks accuracy [8].

Intratumoral heterogeneity is associated with prognosis [9, 10]. A study showed a positive relationship of tumor heterogeneity with recurrence-free survival in breast cancer patients [11]. Nowadays, some methods to evaluate tumor heterogeneity on radiologic images are widely available. Texture analysis (TA) is a tool that has recently gained much attention in the scientific community. In TA, the characteristics of the spatial distribution of pixels and their gray-scale intensities within an image are defined and it has been used in breast tumor studies [12].

Although pretreatment MRI is used in many studies to predict the possibility of achieving pCR after NAT, post-NAT MRI is more useful in clinical practice for determining the presence of residual tumor tissue and for providing critical information for surgical planning. In this study, TA features were extracted from both early and late phases of dynamic contrast-enhanced (DCE) breast MRI after NAT and were used to evaluate the ability of TA to predict whether pCR can be achieved.

## 2. Materials and Methods

### 2.1. Patients

Informed patient consent was waived by the local institutional review board owing to its retrospective design. Between January 2015 and March 2016, 241 consecutive patients with biopsy-proven breast cancers and received treatment at our hospital were filtered by the following criteria. Inclusion: *i.* treatment was NAT followed by surgery; *ii.* completion of both pretreatment and preoperative dynamic contrast-enhanced MR imaging at our institution. Exclusion: *i.* time interval between final MRI examination and date of surgery was longer than 2 weeks (*n* = 18); *ii.* the lesion was reported on pretreatment MRI as a nonmass enhancement (*n* = 64); and *iii.* poor MR image quality or incomplete images retrieved from PACS (*n* = 8). Totally, 151 patients were enrolled and divided into two independent sets on the date of pretreatment MRI: 112 patients with earlier examination time were used as the training set, and the remaining 39 patients were used as the validation set.

### 2.2. MRI Protocols

1.5 T scanner (360; GE Medical Systems) with a dedicated bilateral 4-channel phased-array breast coil was used. Patients were in the prone position. Sequences: (a) fast spin-echo T2-weighted sequences and (b) 3D dynamic contrast-enhanced sequence (VIBRANT) with the following parameters: Sagittal, TR/TE, 5.3 msec/2.6 msec; TI, 12.0 msec; flip angle, 120°; FOV, 20–22 cm; section thickness/gap, 2 mm/0 mm; matrix size with dimensions of 256 × 256; NEX, 0.75; acquisition time for single phase, 45–55 sec with 10–15 sec interval between phases; and total phases acquired, 1 precontrast plus 5 postcontrast series, starting at the same time as contrast injection. A 0.1 mmol/kg bolus of the gadolinium contrast agent was injected into the arm using high-pressure injectors at a rate of 2.0 ml/s followed by a 10 ml saline flush.

### 2.3. MRI Assessment

**Figure 1:**
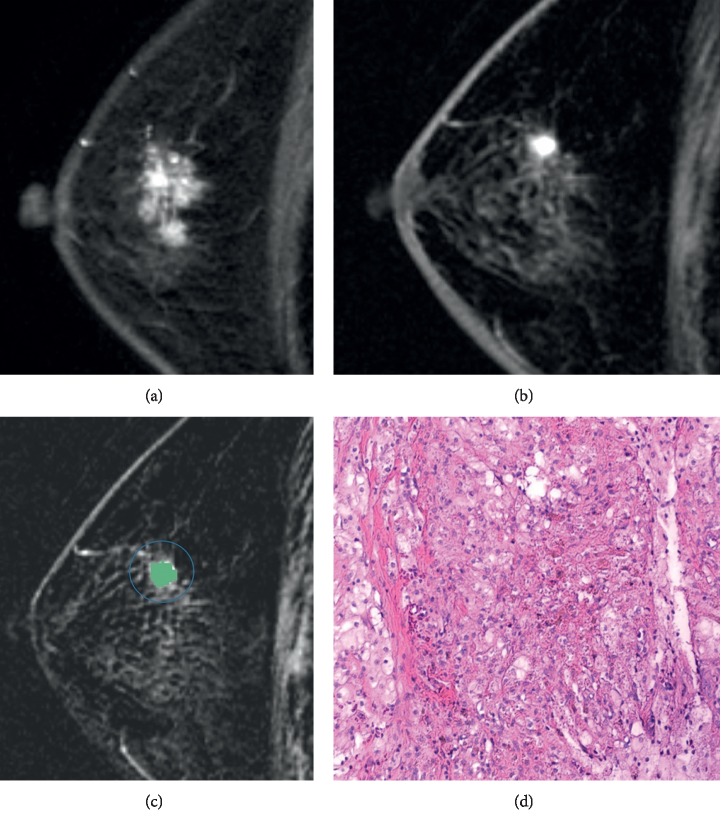
A 62-year-old female with biopsy diagnosed invasive ductal cancer (grade II) in the right breast. MRI showed cancer shrinking from pre- (a) to post-NAT (b). ROIs were drawn on subtracted images (c). It was defined as non-iCR by radiologists. Formula with TA features classified it as iCR (*Y* < 0). Operative pathology revealed scattered fibrosis and lymph cell infiltration in accordance with post-NAT changes. No cancer cells were found (d; as shown by HE staining with a 100x magnification).

Images were retrieved from the local picture archiving and communication system. Pretreatment and post-NAT images were placed side-by-side to locate the exact tumor beds. In cases with multiple lesions, the largest lesion was selected for indexing. On post-NAT early-phase subtracted images (i.e., the subtraction images of the second DCE phase from the precontrast phase), regions of interest (ROIs) were manually placed slice-by-slice to cover the areas with suspicious tumor bed enhancement (Figure 1). For those images with no suspicious enhancement, radiologists were requested to put very small ROIs (four to eight pixels) on the corresponding areas. Late-phase subtractive images were the subtraction images of the last phase from the precontrast phase.

ROIs for the first 30 cases (sorted by MRI date) were drawn, respectively, by a junior resident (Z.B., with 3 years of experience in MRI) and a senior attending (C.K., with more than 10 years of experience in breast imaging) to test interobserver consistency. All other ROIs were initially drawn by Z.B. and then reviewed by C.K. Subjective judgment of complete disappearing of enhancement was recorded as iCR by these two radiologists, discussed together and reached in consensus. Both radiologists were blinded to clinical information during the data collecting period.

Images and ROIs were all transferred into an in-house radiomics software modified on the 3D-slicer platform. 19 conventional TA first-order features were calculated by using the formulas provided by Aerts et al. [13]. Two sets of features were derived from early- and late-subtracted images, respectively, including mean, median, minimal, maximal, 10 percentile, 25 percentile, 75 percentile, 90 percentile, range, interquartile range, variance, skewness, kurtosis, uniformity, energy, entropy, mean absolute deviation, robust mean absolute deviation, and root mean squared. The first 10 features were signal intensity- (SI-) related values, so were calculated as ratios to the mean value on precontrast sequences using same ROIs (rSI = SI/SIpre) when doing comparison between groups and logistic regression analysis. Volumes of the residual-enhancing areas on post-NAT MR imaging was recorded separately as post-NAT-enhancing volume. Thus, 39 features in total were recorded. Lesions defined as complete response on MRI were classified as iCR and others as non-iCR.

### 2.4. Histopathologic Data

All patients underwent either lumpectomy or mastectomy. Final histopathologic results of surgical specimens were reviewed to determine the existence of residual tumor as residual invasive cancer (non-pCR) and no residual invasive cancer cells (pCR), which was defined as either no cancer cells or ductal carcinoma in situ (DCIS). All were referred to local diseases regardless of lymph node status.

### 2.5. Statistical Analysis

SPSS version 22.0 and MedCalc version 15.0 was used. The Cronbach test was used to test the interobserver agreement. After the Kolmogorov–Smirnov test for data distribution, comparisons between pCR and non-pCR groups were made by an independent-sample Student's *t*-test for normally distributed data and the Wilcoxon rank-sum test for others. Receiver operating characteristic (ROC) and binary logistic regression analyses were used to select features and to generate the formula. Four-fold tables were drawn to calculate diagnostic ability. DeLong's test was used to compare area under the curve (AUC). Cronbach's alpha >0.9 and AUC >0.8 were used as the levels to select features. Bonferroni correction was used for multiple comparisons of radiomics features between pCR and non-pCR groups, *P* < 0.001 suggested statistical significance. *P* < 0.05 was considered to indicate statistically significant differences for other tests.

## 3. Results

Clinical information of the 112 patients in the training set and 39 patients in the validation set is presented in Table 1. The mean interval between pre-NAT MRI and surgery was 10.2 days (range, 1–14 days).

### 3.1. Interobserver Consistency Test for ROIs

Post-NAT-enhancing volume and 13 TA features showed high agreement (Cronbach's alpha >0.9; see Table 2) and were used in the following analysis.

### 3.2. Differences between Groups and Feature Selection Using ROC Curves

Twenty-one out of 39 variables showed significant differences between the two groups (*P* < 0.001; see Table 3). Among them, eight showed AUC >0.8 in ROC curves.

### 3.3. Logistic Regression Analysis for Selecting Features

The above eight variables were entered into binary logistic regression. Entropy from the early phase (*P* < 0.001, Exp (B) = 4.922), post-NAT-enhancing volume (*P*=0.216, Exp (B) = 1.001), and robust mean absolute deviation from the early phase (*P*=0.062, Exp (B) = 0.980) were the final three variables left. Entropy was an independent risk factor.

Accordingly, the following formula was synthesized: *Y* = entropy × 1.594 + post-NAT volume × 0.001 − Robust mean absolute deviation × 0.020–3.8. Lesions can be classified as imaging complete response (iCR) when *Y* < 0 (see Figure 1). AUC of this cohort for diagnosing pCR was 0.81 (95% CI 0.72, 0.88), with an accuracy, sensitivity, specificity, positive predictive value (PPV), and negative predictive value (NPV) of 83.93%, 70.00%, 91.67%, 82.35%, and 84.62%, respectively.

### 3.4. Diagnostic Ability Compared with Radiologists

On post-NAT MRI, 27 cases (21 pCR and 6 non-pCR) were identified as iCR using the no enhancement criterion by two radiologists (Table 4), achieving AUC = 0.72 (95% CI 0.63, 0.80), with the accuracy, sensitivity, specificity, PPV, and NPV of 77.68%, 52.50%, 91.67%, 77.78%, and 77.65%. Comparing the diagnostic abilities of using the above TA formula with radiologists' subjective judgment for identifying pCR status after NAT, the AUC was improved significantly (*P*=0.004). The accuracy (*P*=0.02) and sensitivity (*P*=0.02) were also higher, while the specificity, PPV, and NPV remain at similar levels (*P* > 0.05).

## 4. Formula Tested with the Validation Set

In the validation set of 39 cases, using the above TA formula, four pCR cases were wrongly diagnosed as iCR and two non-pCR cases as non-iCR. All other cases were diagnosed correctly. AUC was 0.80 (95% CI 0.62, 0.97).

## 5. Discussion

In assessing post-NAT pCR in breast cancer, reasons of misdiagnosis by MRI vary [6, 14–16]. In our cohort, more cases were seen with residual enhancement, but no invasive cancer on pathology. In Ko's study [17], mild enhancement was also considered as nonresidual and a diagnostic accuracy rate as high as 89% was reached. However, judgment of mild enhancement is quite subjective. Therefore, we aimed to investigate quantitative values that might aid in the identification of the pCR status.

For those cases falsely diagnosed as iCR, the residual tumor was generally small [6, 18, 19]. Volumes of suspicious areas could be acquired preoperatively by MRI prior to pathologic measurements. In our study, this post-NAT-enhancing volume showed a great difference between the pCR and non-pCR groups and could hence be used as a factor in the final formula.

TA provides parameters to quantify cancer heterogeneity. Uniformity and entropy are the most common ones; high entropy and low uniformity represent high heterogeneity. Few studies on the application of TA in breast cancer have been reported. Uniformity and entropy from T2WI and entropy from contrast-enhanced T1WI have been shown to be associated with recurrence-free survival [11], and entropy may differentiate malignant from benign lesions [20]. In our study, entropy was much lower in the pCR group and was the only independent risk factor in logistic regression, and therefore, has the highest weight in the final formula. Uniformity, however, was excluded because of low consistency between observers.

Residual enhancement on MRI is currently the only accepted criterion for determining residual cancer after NAT, but insufficient evidence has shown the optimal choice of phases in DCE imaging. One study confirmed that early-phase enhancement is still superior in predicting posttreatment residual lesions [21]. Therefore, we defined ROIs on early-phase images. Although all features from early and late phases were included in the following analysis, the two retained in the formula were both from the early phase. This may be explained by the fact that the potential correlation among features singled out the strongest ones. We believe that the early phase in DCE imaging plays a more important role in predicting residual tumor than the late phase.

Using the formula with a combination of factors that reflect heterogeneity, the AUC (by ROC analysis) for pCR diagnosis was 0.81 in the training group and 0.80 in the validation group, as compared to 0.72 by radiologists and with improved sensitivity from 52.50% to 70.00%. TA features did not provide useful information for those non-pCR lesions without obvious enhancement, so the specificity was the same for both methods. We did not find any differences in features from basic histograms, for example, percentiles of SI, kurtosis, and skewness, which also reflect distribution heterogeneity. This was likely due to the combined use of all first-order TA features, which consequently reduced the effects of the basic histograms.

Certain limitations of our study exist. This is a single-institute study, with a limited case number. Further validation will be needed. However, compared to those studies that include numerous features and complicated combinations to achieve 100% accuracy and specificity [22], the final features used in our study are much simpler in clinical use, so we expect high reproducibility of our results. Another point to mention is that our results are only applicable to mass-like lesions because nonmass-enhancement lesions on MRI are generally diffuse and scattered and are more likely to suffer from ROI drawing discrepancies.

In conclusion, TA may help to improve the diagnostic ability of MRI in identifying post-NAT pCR in breast cancer, in which entropy, a first-order feature to depict residual tumor heterogeneity, is an important factor. Compared to the judgment by radiologists, the AUC was improved using TA features with higher sensitivity.

## Figures and Tables

**Table 1:** Clinical characteristics of study population.

Characteristics	Training set (*N* = 112)			Validation set (*N* = 39)		
	All	pCR (*N* = 40)	Non-pCR (*N* = 72)	All	pCR (*N* = 16)	Non-pCR (*N* = 23)
Age at diagnosis (y)	48.3 ± 9.7	48.8 ± 9.6	48.0 ± 9.7	47.5 ± 8.9	48.1 ± 7.8	47.2 ± 9.7

*Histology*						
IDC	108	39	69	39	16	23
Non-IDC	4	1	3	0	0	0

*Tumor grade of IDC*						
1	4	1	3	0	0	0
2	77	28	49	23	8	15
3	27	10	17	16	8	8

*Histopathological status HR*						
Negative	33	19	14	15	8	7
Positive	79	21	58	24	8	16

*HER2*						
Negative	69	16	53	24	6	18
Positive	43	24	19	15	10	5

*Pathologic T stage at surgery*						
0	26	26	—	12	12	—
In situ	14	14	—	4	4	—
1	58	—	58	18	—	18
2	9	—	9	3	—	3
3	2	—	2	1	—	1
4	3	—	3	1	—	1

**Table 2:** Interobserver agreement for ROI drawing (Cronbach's test).

Features	Cronbach's alpha	95% confidence interval	*F* value	*P*
Post‐NAT enhancing volume	0.983^*∗*^	0.963, 0.992	57.30	<0.001
Interquartile range	0.965^*∗*^	0.927, 0.983	28.78	<0.001
Skewness	0.410	−0.240, 0.719	1.70	0.081
75 percentile	0.972^*∗*^	0.941, 0.987	35.40	<0.001
Uniformity	0.815	0.612, 0.912	5.41	<0.001
Median	0.938^*∗*^	0.870, 0.971	16.21	<0.001
Energy	0.997^*∗*^	0.995, 0.999	396.06	<0.001
Robust mean absolute deviation	0.969^*∗*^	0.935, 0.985	32.14	<0.001
Mean absolute deviation	0.972^*∗*^	0.940, 0.986	35.14	<0.001
Maximum	0.993^*∗*^	0.985, 0.997	142.62	<0.001
Root mean squared	0.959^*∗*^	0.913, 0.980	24.28	<0.001
90 percentile	0.982^*∗*^	0.961, 0.991	54.52	<0.001
Minimum	−0.140	−1.395, 0.457	0.88	0.637
Entropy	0.972^*∗*^	0.941, 0.987	35.57	<0.001
Range	0.989^*∗*^	0.976, 0.995	88.52	<0.001
Variance	0.967^*∗*^	0.931, 0.984	30.66	<0.001
10 percentile	0.725	0.422, 0.869	3.64	<0.001
Kurtosis	0.877	0.743, 0.942	8.16	<0.001
25 percentile	0.857	0.700, 0.932	7.01	<0.001
Mean	0.944^*∗*^	0.883, 0.974	18.02	<0.001

^*∗*^Features with Cronbach's alpha >0.9.

**Table 3:** Comparing features in pCR and non-pCR groups.

	pCR (*n* = 40)	Non-pCR (*n* = 72)	*Z*/*t*	*P*	AUC (95% CI)
Post‐NAT enhancing volume (mm^3^)	176.05 (31.05, 321.05)	1704.15 (1010.78, 2397.53)	−5.799	<0.001	0.83 (0.75–0.91)^#^

*Early phase*					
Energy	12.18 × 10^6^ (0.58 × 10^6^, 25.77 × 10^6^)	233.12 × 10^6^ (121.26 × 10^6^, 344.97 × 10^6^)	−5.854	<0.001	0.84 (0.76–0.91)^#^
Entropy	2.49 (2.11, 2.87)	4.11 (3.87, 4.35)	−5.902	<0.001	0.84 (0.76–0.92)^#^
Mean absolute deviation	64.29 (47.58, 80.99)	126.09 (110.88, 141.31)	−5.350	<0.001	0.81 (0.72–0.90)^#^
Robust mean absolute deviation	39.36 (25.90, 52.83)	87.41 (75.75, 99.07)	−5.322	<0.001	0.81 (0.71–0.90)^#^
Root mean squared	267.75 (213.47, 318.03)	466.01 (417.13, 514.89)	−5.113	<0.001	0.80 (0.70–0.88)
Variance	9.79 × 10^3^ (4.66 × 10^3^, 14.92 × 10^3^)	30.62 × 10^3^ (23.73 × 10^3^, 37.50 × 10^3^)	−5.447	<0.001	0.81 (0.72–0.90)^#^
rSI_mean_^*∗*^	0.68 (0.56, 0.81)	0.93 (0.84, 1.02)	−3.226	0.002	0.68 (0.58–0.79)
rSI_median_^*∗*^	0.67 (0.55, 0.79)	0.90 (0.81, 1.00)	−3.105	0.002	0.66 (0.56–0.77)
rSI_75 percentile_^*∗*^	0.82 (0.66, 0.98)	1.14 (1.03, 1.26)	−3.416	0.001	0.69 (0.59–0.80)
rSI_90 percentile_^*∗*^	0.94 (0.75, 1.13)	1.39 (1.26, 1.51)	−3.950	<0.001	0.72 (0.62–0.83)
rSI_maximum_	1.09 (0.86, 1.33)	1.91 (1.70, 2.13)	−4.621	<0.001	0.76 (0.67–0.86)
rSI_interquartile range_	0.27 (0.19, 0.35)	0.45 (0.40, 0.51)	−4.172	<0.001	0.74 (0.64–0.84)
rSI_range_	0.74 (0.54, 0.95)	1.67 (1.45, 1.90)	−5.204	<0.001	0.79 (0.71–0.88)

*Delay phase*					
Energy	24.26 × 10^6^ (1.18 × 10^6^, 47.35 × 10^6^)	305.36 × 10^6^ (164.77 × 10^6^, 445.95 × 10^6^)	−5.720	<0.001	0.83 (0.75–0.91)^#^
Entropy	2.77 (2.37, 3.18)	4.35 (4.13, 4.57)	−5.691	<0.001	0.83 (0.74–0.91)^#^
Mean absolute deviation	86.45 (68.58, 104.32)	146.25 (130.89, 161.62)	−4.579	<0.001	0.76 (0.67–0.86)
Robust mean absolute deviation	54.05 (39.26, 68.85)	103.74 (92.29, 115.18)	−4.746	<0.001	0.77 (0.67–0.87)
Root mean squared	373.28 (304.75, 441.81)	576.74 (527.95, 625.52)	−4.755	<0.001	0.77 (0.68–0.87)
Variance	14.78 × 10^3^ (9.05 × 10^3^, 20.50 × 10^3^)	38.54 × 10^3^ (30.43 × 10^3^, 46.65 × 10^3^)	−4.755	<0.001	0.77 (0.68–0.87)
rSI_mean_^*∗*^	0.89 (0.76, 1.03)	1.15 (1.06, 1.24)	−3.242	0.002	0.69 (0.59–0.79)
rSI_median_^*∗*^	0.89 (0.76, 1.02)	1.15 (1.05, 1.24)	−3.254	0.002	0.68 (0.58–0.78)
rSI_75 percentile_^*∗*^	1.07 (0.91, 1.24)	1.42 (1.31, 1.53)	−3.651	<0.001	0.70 (0.60–0.80)
rSI_90 percentile_^*∗*^	1.21 (1.02, 1.41)	1.66 (1.53, 1.78)	−4.026	<0.001	0.73 (0.63–0.83)
rSI_maximum_	1.39 (1.15, 1.64)	2.16 (1.95, 2.36)	−4.445	<0.001	0.75 (0.66–0.85)
rSI_interquartile range_	0.37 (0.28, 0.45)	0.55 (0.49, 0.61)	−3.419	0.001	0.70 (0.59–0.81)
rSI_range_	0.97 (0.73, 1.20)	1.90 (1.67, 2.13)	−4.821	<0.001	0.78 (0.68–0.87)

All data are presented as mean (95% CI); *P* value with Wilcoxon rank-sum test or independent sample *t*-test^*∗*^; ^#^AUC >0.8.

**Table 4:** Diagnostic tables by radiologists and by TA.

		MRI by radiologist		MRI by texture analysis		Total
		iCR	Non-iCR	iCR	Non-iCR	

Pathology	pCR (*n* = 40)	21	19	28	12	
	Non-pCR (*n* = 72)	6	66	6	66	
	Sum	27	85	34	78	112

## Data Availability

Data are available from the corresponding author on reasonable request.

## Abbreviations

AUC: Area under the curve
CI: Confidence interval
DCE: Dynamic contrast enhanced
DCIS: Ductal carcinoma in situ
iCR: Radiological complete response
MRI: Magnetic resonance imaging
NAT: Neoadjuvant therapy
NPV: Negative predictive value
PPV: Positive predictive value
pCR: Pathologic complete response
ROC: Receiver operating characteristic
ROI: Region of interest
SI: Signal intensity
TA: Texture analysis.
